# The etiopathogenesis of atopic dermatitis: barrier disruption, immunological derangement, and pruritus

**DOI:** 10.1186/s41232-017-0044-7

**Published:** 2017-06-05

**Authors:** Pawinee Rerknimitr, Atsushi Otsuka, Chisa Nakashima, Kenji Kabashima

**Affiliations:** 1 Department of Dermatology, Kyoto University Graduate School of Medicine, 54 Shogoin-Kawara, Sakyo, Kyoto, 606-8507 Japan; 2 Division of Dermatology, Department of Medicine, Faculty of Medicine, Skin and Allergy Research Unit, Chulalongkorn University, Bangkok, Thailand; 3 Singapore Immunology Network (SIgN) and Institute of Medical Biology, Agency for Science, Technology and Research (A*STAR), Biopolis, Singapore

**Keywords:** Atopic dermatitis, Immunology, Pathogenesis, Innate, Adaptive, Filaggrin

## Abstract

Atopic dermatitis (AD) is a common chronic skin inflammatory disorder characterized by recurrent eczema accompanied by an intractable itch that leads to an impaired quality of life. Extensive recent studies have shed light on the multifaceted pathogenesis of the disease. The complex interplay among skin barrier deficiency, immunological derangement, and pruritus contributes to the development, progression, and chronicity of the disease. Abnormalities in filaggrin, other stratum corneum constituents, and tight junctions induce and/or promote skin inflammation. This inflammation, in turn, can further deteriorate the barrier function by downregulating a myriad of essential barrier-maintaining molecules. Pruritus in AD, which may be due to hyperinnervation of the epidermis, increases pruritogens, and central sensitization compromises the skin integrity and promotes inflammation. There are unmet needs in the treatment of AD. Based on the detailed evidence available to date, certain disease mechanisms can be chosen as treatment targets. Numerous clinical trials of biological agents are currently being conducted and are expected to provide treatments for patients suffering from AD in the future. This review summarizes the etiopathogenesis of the disease and provides a rationale for choosing the novel targeted therapy that will be available in the future.

## Background

Atopic dermatitis (AD) is a chronic inflammatory skin disorder that affects one fifth of the population in developed countries [1]. The disease is characterized by recurrent eczema accompanied by a chronic intractable itch that leads to an impaired quality of life [2–4]. The onset of AD occurs primarily in childhood and is thought to precede allergic disorders mediated by an immunoglobulin E (IgE) sensitization to environmental antigens, namely, asthma and allergic rhinoconjunctivitis, the so-called atopic march [5–8]. Moreover, there are increasing evidence that AD is associated with systemic diseases and may be considered as a systemic disorder [9, 10]. The prevalence of AD in children is 15 to 25% [11]. Seventy percent of patients outgrow during late childhood [12]. However, many remain affected [13, 14], and some may experience a new disease onset in adulthood [1].

Though extensive recent studies have shed light on the understanding of AD, the exact pathogenesis of the disease remains obscure. The complex interplay among genetics, environmental factors, microbiota, skin barrier deficiency, immunological derangement, and possibly autoimmunity contributes to the development of the disease [15–17]. This review aims to summarize the current understanding of the pathogenesis of AD with a focus on the major etiopathogenesis: barrier disruption, immunological derangement, and pruritus.

## Skin barrier disruption

### Stratum corneum and tight junction

The skin serves as a barrier to protect the body from outside dangers, such as microbes and toxic substances. The epidermis includes four main layers: the stratum corneum (SC), stratum granulosum (SG), stratum spinosum, and stratum basale. SC is the outermost part of the epidermis and is composed of denucleated corneocytes embedded in intercellular lipids (often called “bricks and mortar”) [18], whereas tight junctions (TJs) are intercellular junctions that regulate the paracellular transport of water and solutes [19]. Skin barrier disruption in AD occurs as a result of an aberration of both components.

SC homeostasis relies greatly on filaggrin (FLG) and its metabolic process. The term filaggrin is short for “filament-aggregating protein,” indicating that it is a protein that binds with keratin intermediate filaments and is responsible for the integral structural component [20]. FLG is formed and stored as profilaggrin polymers in keratohyalin granules in SG. At the interface between SG and SC, profilaggrin polymers are cleaved into FLG monomers by certain proteases, such as CAP1 [21] and SASPase [22, 23]. These monomers then assemble with keratin intermediate filaments to strengthen SC. Finally, at the upper SC, FLG is degraded into amino acids, urocanic acid (UCA), and pyrrolidine carboxylic acid (PCA). UCA is responsible for the acid mantle of the skin, and PCA provides natural moisturizing factors in the skin. This degradation process is mediated by proteases, namely, caspase 14 [24], calpain 1 [25], and bleomycin hydrolase [25].

Networks of TJs are found in the intercellular spaces of SG and regulate the paracellular transport of water, ions, and solutes [19, 26]. Strands of TJs are composed of the transmembrane portion in which claudins and occludins represent the most abundant constituents. Zonula occludens (ZO) is the major cytosolic scaffolding proteins responsible for the TJ assembly [27].

The importance of TJs in barrier function has been clearly demonstrated: mice with claudin-1 deficiency die within a day after birth with wrinkling skin [28]. Importantly, TJ aberration is associated with AD since human AD epidermis has a reduced expression of claudin-1, claudin-23 [29], and ZO-1 [30] and shows evidence of impaired barrier function. In addition, polymorphisms of *CLDN1*-encoding claudin-1 are found in AD patients [29].

### Filaggrin and its role in the pathogenesis of AD

FLG is essential for controlling transepidermal water loss and maintaining SC hydration [31, 32] and for the cornification and organization of the epidermis [31, 33]. FLG is known to be decreased in the epidermis of AD patients [34], and null mutation in FLG is the strongest risk factor for AD [35]. FLG haploid insufficiency further confers the risk of developing of several atopic diseases, including asthma, food allergy, and allergic rhinitis [36].

FLG deficiency also leads to an increase in skin pH, which, in turn, enhances the function of serine proteases kallikrein (KLK)5, KLK7, and KLK14, which are responsible for corneocyte shedding [37]. These activated KLKs can increase the production of interleukin (IL)-1α and IL-1β from corneocytes [38]. Moreover, by binding to the protease-activated receptor type 2 (PAR2) on keratinocytes, KLKs can induce thymic stromal lymphopoietin (TSLP) production that further promotes inflammation [39, 40].

### The analysis with Flg mutant mice

The importance of FLG in the pathogenesis of AD is supported by the evidence that mice with Flg deficiency, e.g., flaky tail (*Matt* ^*ma*/*ma*^ *Flg* ^*ft*/*ft*^) mice and Flg mutant (*Flg* ^*ft*/*ft*^) mice on a proallergic BALB/c background, exhibit a spontaneous AD phonotype [7, 41–43]. Of note, the flaky tail mice harbor double gene mutations, *Flg* and matted (*ma*), both of which affect the skin barrier in a different fashion. Abnormality in *Flg* leads to an aberrant profilaggrin polypeptide expression while *ma* mutation gives rise to the matted hair and spontaneous dermatitis phenotype [41, 44]. In addition, the derangement in proteases required for profilaggrin and Flg processing also gives rise to impairment of the skin barrier and SC dehydration, as observed in mice deficient in CAP1 [21], SASPase [22], and caspase 14 [24].

Furthermore, increased allergen penetration is observed in Flg-deficient mice, e.g., Flg-null mice [45] and flaky tail mice [46], and augmented responses in contact hypersensitivity are detected [45]. Allergen penetration results in inflammasome and protease activation [47]. Additionally, a reduction in FLG predisposes microbial colonization in the skin [32], partly due to the loss of the acid mantle resulting from the decrease in FLG breakdown products [48] combined with the indirect neutralizing effects of FLG to α-toxin of *Staphylococcus aureus* [49]. This effect is known to be mediated by the secretion of sphingomyelinase, an enzyme stored in the lamellar bodies of keratinocytes in which FLG is required for proper secretion [49–51]. Intriguingly, by promoting Flg expression in NC/Nga mice, development of the AD phenotype in the mice is attenuated, and upregulating FLG may be one of the approaches to improve AD [52].

**Fig. 1:**
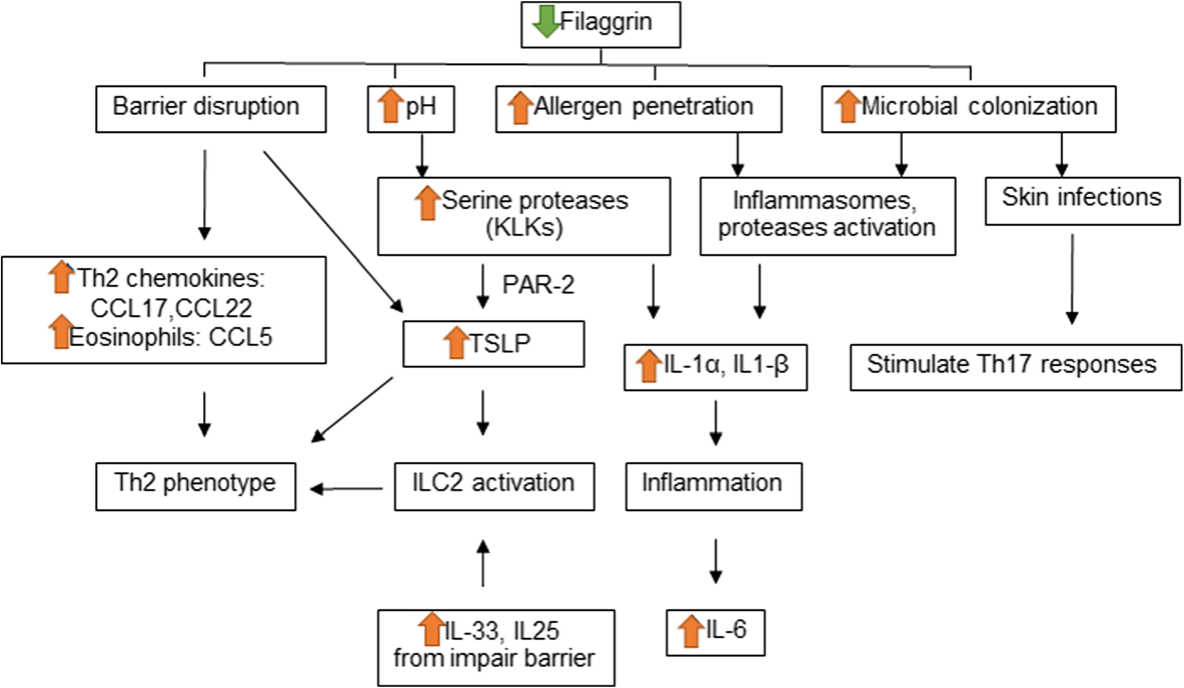
Immunologic modulation of filaggrin (FLG) in the development of atopic dermatitis. Decreased FLG exacerbates skin inflammation in many ways. Th2 phenotype skewing occurs because of barrier disruption and keratinocyte injuries that stimulate thymic stromal lymphopoietin (TSLP), Th2, and eosinophil-recruiting chemokines together with IL-33 and IL-25 released from keratinocytes. Moreover, the loss of the acid mantle in the epidermis also promotes TSLP secretion via protease-activated receptor type 2 (PAR-2) activation by increased serine proteases. Enhanced allergen penetration and microbial colonization activate inflammasomes and the Th17 pathway that complicate the pathogenesis of AD in a later state

Sweating is known to be attenuated in AD, which may be a result of acrosyringium obstruction caused by abnormalities in the sweat duct structures and/or derangement in the common sudomotor nerves that control sweating [53, 54]. Recently, it has been demonstrated that a sweat duct obstruction is observed in Flg mutant mice and that sweating is consequently reduced. These findings suggest that FLG may also contribute to the integrity of the acrosyringeal wall [55]. The immunologic modulation of FLG in the development of AD is summarized in Fig. 1.

### Other SC components and their relationship with AD

Intercellular lipids are a fundamental part of SC and are regarded as the mortar in the brick and mortar model of the epidermis. These lipids are composed of ceramides, free fatty acids, and cholesterol in a ratio of 1:1:1 M [18]. The lipid precursors are formed and stored in the lamellar bodies of SG and are released into the extracellular space when the keratinocytes differentiate into SC [18]. Abnormalities in the enzymes responsible for lipid processing and the transportation of lamellar bodies across the cells give rise to a myriad of barrier-insufficient skin diseases. For example, mutations of the genes that encode the enzymes 12R-lipoxygenase and epidermal lipoxygenase 3 are associated with autosomal recessive congenital ichthyosis (ARCI) [56]. It is of note that transmembrane protein 79/mattrin (Tmem79/Matt), a five-transmembrane protein of lamellar bodies, is essential in the lamellar body secretory system and that flaky tail (*Matt* ^*ma/ma*^ *Flg* ^*ft/ft*^) mice and Tmem79 (*ma/ma*) mice with *ma* mutation exhibit spontaneous AD-like dermatitis [44, 57]. Moreover, in the human counterpart, a meta-analysis revealed that a missense mutation in the human *MATT* gene is associated with AD [57].

Corneocyte shedding is tightly regulated by serine proteases and serine protease inhibitors as mentioned above. Serine protease inhibitors include lymphoepithelial Kazal-type 5 serine protease inhibitor (LEKTI), which is encoded by the serine protease inhibitor Kazal-type 5 (*SPINK5*) [58]. Disorders involving mutation and genetic polymorphisms of the genes that encode KLKs and LEKTI exhibit AD-like phenotypes [59–61]. For instance, Netherton syndrome is caused by *SPINK5* mutations. Patients with this disease exhibit severe dermatitis, allergic rhinoconjunctivitis, asthma, and a high serum IgE level [60]. Moreover, recent studies show that polymorphisms in *SPINK5* are related to AD [61, 62]. Skin barrier dysfunction and cutaneous inflammation due to aberrant immunologic responses are critical for AD development [1, 63]. The initial trigger, however, remains a subject of debate. Although null mutation of the *FLG* gene poses the strongest risk for AD, 60% of individuals who carry the gene do not have AD symptoms [36]. On the contrary, a significant portion of AD patients do not have *FLG* mutation [36]. It is thus evident that additional factors are needed to develop the disease. In fact, recent genome-wide association studies reported ten new loci that are associated with AD and show a relationship with autoimmune regulation, especially in innate signaling and T cell activation [35, 64]. In the following section, immunological derangement in AD will be discussed.

## The immunopathogenesis of AD

### Keratinocytes

Acute skin barrier disruption promotes T helper (Th)2 skewing. Keratinocyte-derived cytokines such as TSLP [65], which is known to promote the AD-like phenotype [66], IL-25, IL-33, and granulocyte-macrophage colony-stimulating factor (GM-CSF) influence innate lymphoid cells (ILCs) and increase the production of Th2 chemokines: CCL17 (thymus- and activation-regulated chemokine (TARC)), CCL22, and an eosinophil chemoattractant: CCL5 (regulated upon activation, normal T cell expressed and secreted (RANTES)) [67]. In addition to promoting Th2 cell recruitment, CCL17 has been reported to enhance keratinocyte proliferation and implicate in AD development [68].

### ILCs, basophils, eosinophils, and mast cells

ILCs are a novel group of innate immune cells developed from a common lymphoid progenitor [69]. Although the morphology of ILCs resembles that of lymphoid cells, ILCs do not carry an antigen receptor. Instead, they have similar transcription factors as T cell subsets. Therefore, ILCs are capable of producing hallmark cytokines in the same fashion. ILCs can be divided into three groups: ILC1, ILC2, and ILC3. ILC2, characterized by having the transcription factor GATA3 and producing Th2 cytokines (IL-4, IL-5, and IL-13), is considered to be important in the pathogenesis of AD.

**Fig. 2:**
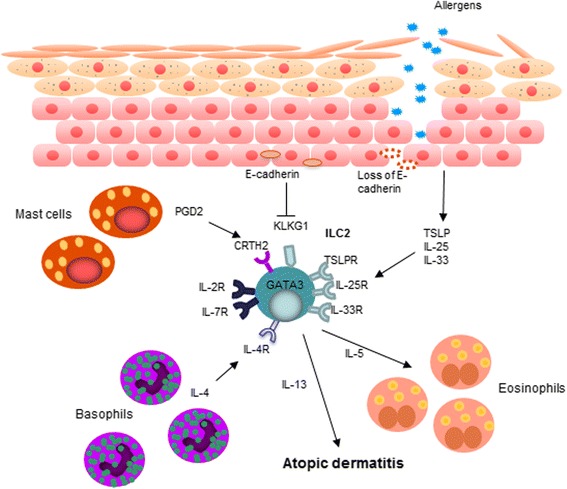
Interplay among the barrier dysfunction, innate lymphoid cell (ILC)2, basophils, eosinophils, and mast cells. Barrier disruption leads to the production and release of epithelial-derived cytokines, namely, thymic stromal lymphopoietin (TSLP), IL-25, and IL-33. Upon ligation with the corresponding receptors on ILC2, TSLP receptor (TSLPR), IL-25 receptor (IL-25R, also known as IL17RB), and IL-33 receptor (IL-33R or ST2), ILC2 is activated to release Th2 cytokines, e.g., IL-5 and IL-13. In addition, IL-4 from basophils which are found in proximity to ILC2 in AD skin lesions can directly activate ILC2. PGD2, presumably from mast cell degranulation, also contributes to the recruitment of ILC2 into the skin as well as the induction of ILC2 Th2 cytokine production. In contrast, cell adhesion molecules, E-cadherin, on keratinocytes, are known to have an inhibitory effect on ILC2. Nevertheless, loss of E-cadherin is observed in FLG-deficient individuals. Therefore, skin inflammation is enhanced as there is an increase in stimulatory but a decrease in inhibitory stimuli

Several studies have shown that, although few ILCs are observed in normal human skin, in AD, the skin is infiltrated markedly by the ILC2 subset [70, 71]. IL-5 and IL-13 released from ILC2 are essential and sufficient to induce AD lesions in mouse models [71, 72]. In addition, ILC2 can drive Th2 responses in vivo [71]. Certain epithelial-derived cytokines and eicosanoids, namely, TSLP [71], IL-25 [73], IL-33 [73, 74], and prostaglandin D2 (PGD2) [75, 76], can activate ILC2 whereas E-cadherin [73] is known to have an inhibitory effect. In addition, ILC2 can also respond to hematopoietic cell-derived cytokines, IL-2 and IL-7 [72]. Importantly, IL-4 from basophils can directly activate ILC2 and bring about AD-like inflammation [77]. The immunomodulatory actions of the aforementioned cytokines are mediated by ligation with the corresponding receptors that are present on the ILC2. In AD, where the epidermal barrier is breached, epithelial-derived cytokines (TSLP, IL-25, IL-33) are released. In addition, FLG deficiency that leads to a reduction in E-cadherin [73, 78] together with an increase in IL-4 derived from basophils and PGD2 from mast cells is present. These environments promptly recruit and activate ILC2 and initiate the cutaneous inflammation observed in AD. The complex interplay between the barrier dysfunction, ILC2, basophils, eosinophils, and mast cells is involved in AD [69, 79–82], as shown in Fig. 2.

In addition to cytokines and chemokines, the neurotransmitter dopamine is involved in mast cell activation and Th2 skewing in AD. Mast cells and Th2 cells bear dopamine receptors, the D1-like receptor group. Upon ligand binding, mast cells are degranulated and Th2 cells are activated, observed by an increase in the ratio of IL-4 to IFN-γ mRNA expression [83]. As psychoneuroimmunology plays a role in many skin diseases, results from the study might explain the worsening of AD symptoms after a psychological strain.

### Dendritic cells (DCs)

DCs are professional antigen-presenting cells that capture antigens, allergens, and microbes, to prime naïve T cells into immunogenic or tolerogenic subsets, and act as a bridge between innate and adaptive immunity [84]. In the steady state of the skin, Langerhans cells (LCs) reside in the epidermis, and groups of dermal DCs (DDCs) are found in the dermis. It has been suggested that DCs initiate AD in humans, although it remains unclear which cutaneous DC subsets initiate epicutaneous sensitization.

LCs perform surface antigen surveillance and recognition by extending their dendrites through the TJs to take up the antigens [85]. LCs’ function in this regard has been reported to induce Th2 responses as LCs efficiently drive naïve CD4^+^ T cells into Th2 cells [86]. It is speculated that a breach in TJs may enhance penetration of foreign antigens, which are then taken up by LCs, amplifying Th2 cutaneous inflammation.

LCs are also responsible for the initiation of AD under the influence of TSLP [63, 87, 88]. Nakajima et al. demonstrated that LCs are essential to the induction of clinical manifestations and IgE elevation in a mouse model upon epicutaneous sensitization with the protein antigen ovalbumin (OVA) via the action of TSLP and its receptors presented on LCs [87]. TSLP is abundantly expressed in keratinocytes of lesional and non-lesional skin in AD patients as a result of both skin barrier dysfunction and proinflammatory cytokines, such as IL-1β, tumor necrosis factor (TNF)-α, IL-4, and IL-13 in AD [65, 89]. Importantly, TSLP can trigger DC migration to the draining lymph nodes [90], and LCs treated with TSLP drive naïve T cells to Th2 phenotypes [91]. All of these events lead to a Th2 bias in the acute stage of AD.

During the inflammatory state of AD, myeloid inflammatory DCs, namely, inflammatory dendritic epidermal cells (IDECs), are recruited [92]. DCs in the skin of AD patients are well characterized by the presence of a high-affinity receptor for IgE (FcεRI) that renders these DCs to induce T cell responses effectively [92]. A wide spectrum and amount of foreign surface and penetrating antigens, microbes, and allergens are encountered in AD skin due to the breach in the epidermis and the barrier dysfunction [92].

The cross-linking of FcεRI with IgE on the LC surface in an in vitro study leads to a release of chemokines, for example, IL-6, CCL22, CCL17, and CCL2, which can recruit Th2 cells and other immune cells, importantly, IDECs [93]. A patch test study showed that IDECs rapidly migrate into the epidermis of patch-tested lesions [94]. IDECs are key DCs in amplifying skin inflammation as they can migrate to the draining lymph nodes and prime naïve T cell into interferon (IFN)-γ- and IL-18-producing T cells [93]. Therefore, IDECs are considered to be important in the switching of Th2 to Th1 in chronic AD [94].

Another interesting feature of AD is a decreased plasmacytoid DC (pDC) number in AD skin compared to that expected to be observed in skin inflammatory conditions [95]. This can be explained by a lack of chemoattractant for pDCs, i.e., chemerin, in AD skin [96] and a paucity of skin-homing molecules in the blood pDCs of AD patients [97] in combination with the Th2 milieu in the skin [84]. pDCs are crucial in immune responses against viral infections. The reduced number and improper function of pDCs observed in AD skin can thus contribute to a susceptibility to viral infections [97].

### Th2/Th1 paradigm shift

AD is traditionally viewed as a Th2-mediated allergic disease with increased IgE production, eosinophilia, mast cell activation, and overexpression of Th2 cytokines IL-4, IL-5, and IL-13 [63, 98]. Th2 polarization and barrier defects are closely related. TSLP, IL-25, and IL-33 are upregulated in the epithelium after environmental signals [11]. Keratinocytes are the main source of TSLP, which is crucial in the induction of Th2 skewing in AD skin by the activation of LCs and DCs [99]. In turn, Th2 cytokines IL-4 and IL-13 can induce keratinocytes to express TSLP [100]. In addition, Th2 cytokines can inhibit the expression of Toll-like receptors (TLRs), which dampens host defense against infections [100]. Moreover, IL-4 and IL-13 have negative impacts on skin barrier function. FLG, loricrin, and involucrin, the integral components of SC, are downregulated with Th2 cytokines, regardless of FLG genotypes [101, 102]. Keratinocyte differentiation is perturbed by Th2 cytokines via STAT3 and STAT6 activation, and topical Janus kinase (JAK) inhibitors can restore epithelial function by suppressing STAT3 signaling [103]. B cell class switching and increased IgE synthesis are also induced by IL-4. Based on this supporting evidence, suppression of Th2 cytokines appeared to be useful in alleviating AD symptoms [104–106]. Recently, a novel biological agent, the anti-IL-4 receptor antibody dupilumab, has been studied in clinical trials and found to be promising for the treatment of AD [107].

### Th17

Th17 cells have the capacity to produce IL-17A, IL-17F, IL-22, and IL-26 upon stimulation [108]. Indeed, IL-17 can induce S100 protein and proinflammatory cytokine expression, which are responsible for eosinophil- and neutrophil-mediated inflammation [109, 110]. Many studies have suggested the involvement of Th17 in the pathogenesis of AD. For instance, it is reported that epicutaneous sensitization in mice with OVA typically leads to AD-like dermatitis and the cutaneous expression of IL-17 and IL-17-producing T cells in the draining lymph nodes and spleen, as well as increased serum IL-17 levels [111]. In addition, a study that used a repeated hapten application to induce AD in mice showed that IL-17A is necessary for the development of skin inflammation, IL-4 production, and IgG1 and IgE induction [112]. Interestingly, IL-17A is detected in the AD-like dermatitis of flaky tail mice [42, 46, 113]. Consistently, analysis of the peripheral blood of severe AD patients reveals an increased number of IL-17-producing cells [114, 115].

AD can be divided into two types: extrinsic and intrinsic AD. Patients with extrinsic AD typically have an elevated IgE level, harbor *FLG* mutation with a disruptive barrier, exhibit an early onset, and traditionally have a Th2-dominant response. On the other hand, intrinsic AD patients exhibit different features [116–118]. Patients with intrinsic AD usually do not show an elevated IgE level, do not harbor *FLG* mutation, exhibit with an adult onset, and are associated with more Th17 and Th22 immune activation than extrinsic AD patients [119]. Essentially, a study that compared phenotypes between European/American versus Asian AD showed that Asian AD skin exhibits more epidermal hyperplasia and parakeratosis that skewed toward psoriasis features. It is of note that higher Th17 activation is observed in Asian AD; this needs to be considered in the selection of treatments for different populations [120].

### Antimicrobial peptides

Atopic skin is characterized by increased *S. aureus* colonization and/or infections with a loss of microbial diversity during flares [121]. This may be explained partly by the reduction in antimicrobial peptides (AMPs) of the skin. In homeostasis, AMPs, such as S100 protein psoriasin, ribonuclease (RNase) 7, dermcidin, and lactoferrin, are constitutively present in the epidermis and serve as one of the first-line protections against microbes. Upon challenges with danger signals/organisms, additional AMPs, namely, human beta-defensins (hBD)-2, hBD-3 and cathelicidin (LL-37), are upregulated [122]. In the past, it was thought that the reduction in hBD-2, hBD-3, and LL-37 levels in AD epidermis was one of the causes of *S. aureus* overgrowth [123]. This can be explained by the fact that skin inflammation in AD is predominantly mediated by Th2 cytokines (IL-4, IL-13) in the acute phase of the disease [63] and that the Th2 cytokine milieu is known to suppress the production of the AMPs [124]. Recent findings, however, have shown that the induction of the AMPs hBD-2, hBD-3, and LL-37 was not impaired in AD [125]. These productions may not be sufficient to tackle the infections or the AMP functions may be disturbed [126]. Interestingly, *Escherichia coli* infection is rarely encountered in AD, and the major AMP family that combats this microbe is the S100 proteins, which consist of S100A7 (psoriasin), S100A8, and S100A9 [124]. An increase in S100 proteins in patients with AD has been reported [124, 125]. In addition, S100 proteins have proinflammatory properties that can further promote skin inflammation and inhibit keratinocyte differentiation, complicating the barrier dysfunction [126, 127].

Recent interesting studies have shown that TSLP exists in two different isoforms, i.e., short and long forms, both of which exhibit different functions. In the skin and gut, the short form is constitutively expressed, which is responsible for maintaining tissue homeostasis and acts as antimicrobial peptides [128, 129]. In contrast, the long form predominates and possesses proinflammatory activity under inflammatory states, such as in the lesional skin of AD [128, 130].

Pattern recognition receptors (PRRs) recognize vital and highly conserved molecular structures of microorganisms, so-called pathogen-associated molecular patterns (PAMPs), and danger signals, damage-associated molecular patterns (DAMPs). Upon receptor ligation, signaling pathways are stimulated and result in the production of cytokines with biological effects. In the skin, PRRs are found in keratinocytes and other innate immune cells [131] and are known to be involved in the pathogenesis of AD. TLRs are the most well-characterized PRRs. Other PPRs include NOD-like receptors (NLRs), RIG-I-like receptors (RLRs), and C-type lectin receptors (CLRs).

TLR2 is a major ligand for *S. aureus* [132] that has a unique ability to form either a homodimer or a heterodimer with TLR1 or TLR6 to expand their binding spectrum capability [133]. Bacterial lipoteichoic acid (LTA) found in *S. aureus*-infected AD lesions can bind to TLR2, which then exerts immunologic responses. Interestingly, a single nucleotide polymorphism (SNP) of TLR2 has been reported to be associated with severe AD and implicated in an increase in *S. aureus* infections [134, 135]. Moreover, intracellular muramyl dipeptide (MDP) derived from *S. aureus* peptidoglycan is recognized by NOD2, NLR family, within keratinocytes [136]. It is of note that NOD2-deficient mice exhibit an increased susceptibility to subcutaneous *S. aureus* infection [137], and polymorphisms in the NOD2 gene are associated with human AD [138].

Interestingly, peptidoglycan from *S. aureus* induces signaling through NOD-2 coupled with TLR stimulation which efficiently induces DCs to produce IL-12p70 and IL-23, which drive Th1 and Th17 responses, respectively [139]. This may in part explain the shift of Th2-dominated to Th1/Th17-co-dominated inflammation in the chronic stage of AD where multiple PRRs are engaged [133].

### *S. aureus* and AD

The cutaneous microbiome plays an important role in the skin during homeostasis and in the disease state. This is especially evident in AD in which skin colonization with *S. aureus* is found in almost 90% of lesional skin and 55 to 75% of non-lesional skin [5]. Skin infections and the toxin from *S. aureus* further exacerbate the cutaneous proinflammatory state [140]. Importantly, skin colonization with *S. aureus* produces superantigens that also stimulate Th17 responses by increasing IL-17 production [140, 141]. Indeed, in AD patients with skin infections, a rise in the percentage of Th17 cells in peripheral blood has been found [142]. *S. aureus* can worsen barrier impairment [143]; releases proteases, enzymes, and cytolytic toxins that induce cell injuries [143, 144]; and engages PRRs and exerts inflammation [145]. Its toxic shock syndrome toxin 1 (TSST-1) and enterotoxins (SEs) act as superantigens to activate a large number of T cells [146], promote T cell recruitment to the skin [147], and exert superantigen-specific IgE [148]. Additionally, *S. aureus* δ-toxin can stimulate mast cell degranulation [149]. These summarized events eventually deteriorate the disease state of AD [150].

## Pruritus

Itch or pruritus is one of the most disturbing symptoms that characterize AD and can in fact significantly impair the quality of life of affected individuals [151]. Pruritus in AD is a result of the complex interplay among many factors. Although the exact pathogenesis remains unknown, recent studies have shown that hyperinnervation of the epidermis, an increase in several itch mediators/pruritogens, and central sensitization of itch are evident in AD.

### Hyperinnervation of AD skin

The sprouting of nerve fibers is under a balanced homeostasis between nerve sprouting factors, e.g., nerve growth factor (NGF), amphiregulin, and gelatinase versus nerve retraction factors, e.g., semaphorin 3A (Sema3A) and anosmin-1 [152]. An increase in nerve fiber density in the epidermis is reported in AD [153, 154]. This is partly explained by the elevation of NGF that is observed in the plasma of AD patients [155] and a decrease in Sema3A that is detected in the lesional AD epidermis [156].

In addition to the hyperinnervation of the skin, a lower threshold for activation of the sensory nerve fibers is also observed in AD, and these events mutually function to increase the excitability of the sensory nerves [157]. This hypersensitivity of the primary itch-sensing neurons may contribute to alloknesis, pruritus resulting from non-pruritogenic stimuli [158], which is a well-observed phenomenon in AD patients [159]. Interestingly, artemin, a glial cell-derived neurotrophic factor (GDNF)-related family, is reported to be important in warm inducing itch because artemin is upregulated in fibroblasts from AD lesions, and an intradermal injection of artemin in mice leads to an increased number and sprouting of peripheral nerves together with thermal hyperalgesia [160, 161].

### Itch mediators/pruritogens

Several itch mediators and their corresponding receptors are reported to be responsible for itch in AD: histamine (H), especially the role of H1 and H4 receptors (H1R and H4R) [157, 162], certain proteases (including tryptase, dust mites, and *S. aureus* [151]), substance P [63, 151], IL-31 [163, 164], TSLP [165], and endothelin-1 [166].

Recently, much interest has been focused on the role of IL-31-induced pruritus. IL-31 is predominantly produced by Th2 cells, and its receptor, which comprises IL-31 receptor α (IL-31RA) and oncostatin M receptor β (OSMRβ), is expressed in peripheral nerve fibers, dorsal root ganglions (DRGs), and keratinocytes [167, 168]. Upon its ligand binding, IL-31 signaling is mediated by activation of the JAK-signal transducer and activator of transcription (STAT) (STAT-1/5 and ERK-1/2), mitogen-activated protein kinase, and phosphoinositide-3-kinase (PI3K) signaling pathways [169, 170]. The IL-31 level is elevated in AD skin as well as in the serum [163, 171]. The IL-31 serum level correlates with the disease severity [172]. Clinical trials related to anti-IL-31 receptor (nemolizumab) and anti-IL-31 (BMS-981164) as treatments for AD are currently being conducted [173].

### Central sensitization of pruritus in AD

The role played by the central nervous system in terms of the itch associated with AD has been investigated to a lesser extent compared to that of peripheral innervation. A study that used functional magnetic resonance imaging with arterial spin labelling, however, showed an increase in the activation of the anterior cingulate cortex and dorsolateral prefrontal cortex in human AD subjects compared with that of healthy controls [174]. These results suggest a central sensitization in AD individuals. Importantly, cognitive and affective processes play a pivotal role in the interpretation and perception of pruritus [151]. This is evident in AD, as several psychotropic drugs, including antidepressants, can attenuate the itch severity in some patients [175].

In addition to the brain itself, the activation of STAT3 in the astrocytes of the spinal dorsal horn has been shown to be involved in chronic pruritus. This activation results in the production of lipocalin-2 that enhances pruritus and may lead to a vicious itch-scratch cycle [176]. It is of note that astrocytes are a subtype of glial cells of the central nervous system [151].

**Fig. 3:**
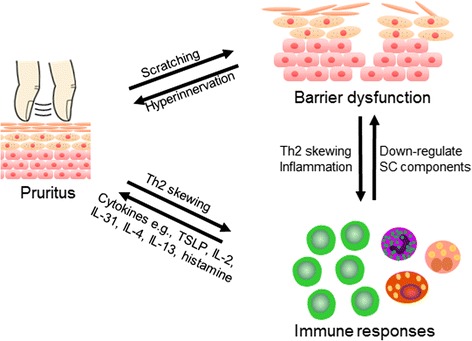
The relationship among itch, barrier disruption and an immunologic aberration. Scratching resulting from itch can worsen a breach in the skin. Dry skin promotes itch by increasing the density of epidermal nerve fibers. Scratching also promotes Th2 chemokines, eosinophil-recruiting chemokines, and thymic stromal lymphopoietin (TSLP). Conversely, immune responses can induce itch via the secretion of a myriad of cytokines that can act as pruritogens

The complex interplay among itch, barrier disruption, and immunologic aberration is illustrated in Fig. 3. Pruritus is known to induce scratching behavior that introduces or worsens breaches in the skin. On the other hand, in dry skin model mice, epidermal barrier dysfunction is observed together with an increase in the number of epidermal nerve fibers [177]. With regard to the relationship between pruritus and the immune responses, once the barrier is disrupted from scratching, e.g., in an experimental tape stripping procedure, Th2 chemokines (CCL17 and CCL22) and eosinophil-recruiting chemokines (CCL5) are increasingly produced by keratinocytes [67]. Moreover, tape stripping also results in TSLP production in the skin [65]. Consequently, Th2 skewing ensues. Conversely, immune responses can induce itch via the secretion of a myriad of cytokines that can act as pruritogens, namely, TSLP, IL-2, IL-31, IL-4, and IL-13 [157].

## Novel AD treatments

Based on the growing knowledge of the complex pathophysiology of AD, many novel targeted therapies are currently in clinical trials. Table 1 summarizes these novel treatments [11, 173, 178–180].

**Table 1:** Novel AD treatments. The table is modified from Heratizadeh and Werfel [178], Lauffer and Ring [179], Noda et al. [180], Nomura and Kabashima [173], and Werfel et al. [11]

Mechanism of action	Route	Compound	Company	Type of clinical trial	Result
Targeting pathogenic cytokines and their cognate receptors					
IL-4/IL-13 receptor α-chain antagonist	SC	Dupilumab	Regeneron	Phase III	Improvement in clinical outcomes, pruritus, and quality of life
IL-13 antagonist	SC	Lebrikizumab	Roche	Phase II	Not yet available
	SC	Tralokinumab	AstraZeneca	Phase II	Not yet available
IL-23p40 antagonist	SC	Ustekinumab	Janssen	Phase II	Not yet available
IL-22 antagonist	IV	Fezakinumab	Pfizer	Phase II	Not yet available
IL-31 receptor antagonist	SC	Nemolizumab	Roche	Phase II	Improvement in pruritus and EASI score
IL-31 antagonist	SC, IV	BMS-981164	Bristol-Myers Squibb	Phase I	Not yet available
IL-1R1 antagonist	SC	Anakinra	Sobi	Phase I	Unpublished
IL-6	SC, IV	Tocilizumab	Genentech	Case series	Improvement in EASI score
Targeting pathogenic molecules					
PDE-4 inhibitors	Topical	Crisaborole	Anacor	Phase III	ISGA score success
	Topical	E6005	Eisai	Phase II	EASI score and SCORAD improvement
	Topical	DRM02	QLT	Phase II	Not yet available
	Oral	Apremilast	Celgene	Phase II	EASI score improvement
CRTh2 antagonist	Oral	ODC-9101	Oxagen	Phase II	Not yet available
	Oral	Fevipiprant	Novartis	Phase II	Not yet available
JAK inhibitor	Topical	Tofacitinib	Pfizer	Phase II	Decrease in EASI score
	Oral	Pf-04965842	Pfizer	Phase II	Not yet available
TSLP antagonist	SC	Tezepelumab	Amgen	Phase II	Not yet available
Targeting IgE					
IgE antagonist	SC	Omalizumab	Novartis	Stopped after proof-of-concept study	Heterogeneous results
	SC	Ligelizumab	Novartis	Phase II	Not yet available

*Abbreviations*: *PDE* phosphodiesterase, *CRTh2* chemoattractant receptor-homologous molecule expressed on Th2 lymphocytes, *JAK*, Janus kinase, *TSLP* thymic stromal lymphopoietin, *SC* subcutaneous injection, *IV* intravenous, *ISGA* Investigator’s Static Global Assessment, *SCORAD* SCORing Atopic Dermatitis, *EASI* Eczema Area and Severity Index

### Anti-IL-4 receptor, dupilumab

Based on the importance of IL-4 in inducing AD inflammation and barrier impairment, studies have examined how to attenuate its function. The central focus with a growing number of evidence is dupilumab, a fully humanized monoclonal antibody against the IL-4 receptor α subunit (IL-4Rα). This subunit is shared by IL-4 and IL-13; therefore, by blocking IL-4Ra, both IL-4 and IL-13 are inhibited [181].

Several phase II studies have shown the efficacy of dupilumab in improving clinical outcomes, biomarkers, and the transcriptome level in AD patients [181–183]. Moreover, a recent phase III study has confirmed the findings in a larger number of patients [184]. Although further studies with long-term follow-up periods are needed, dupilumab appears to be a promising treatment modality for AD.

### IL-13 antagonist

IL-13 belongs to the Th2 cytokine lineage that can bind to both IL-4 and IL-13 receptors; therefore, the functions of IL-4 and IL-13 are considered to be similar [11]. IL-13 is important in B cell activation and differentiation and in promoting IgE production by B cells [178]. Lebrikizumab and tralokinumab are monoclonal antibodies against IL-13. Their roles in AD treatment are currently being explored in current phase II studies (ClinicalTrials.gov identifier: NCT02340234 and NCT02347176).

### IL-23p40 antagonist

The fact that Th1 and Th17 cells are involved in the pathogenesis of AD has led to a trial of ustekinumab in AD. Ustekinumab is a fully human monoclonal antibody against the receptor p40 subunit, shared by IL-12 and IL-23, which are required for the development and maintenance of Th17 and Th1 cells [179]. Notably, a recent phase II study showed no significant differences in clinical efficacy (SCORAD50) between ustekinumab and placebo in adult AD patients, which may be explained by an inappropriate dosing regimen [185]. Another phase II study is being evaluated (NCT01806662).

### IL-31 receptor antagonist and IL-31 antagonist

IL-31 is an important mediator of both itch and inflammation [186, 187]. Nemolizumab, an IL-31 receptor antagonist, has been shown to significantly reduce itch and improve the Eczema Area and Severity Index (EASI) score in AD patients [188]. Additionally, a result from a phase I/Ib study suggests that nemolizumab is well tolerated and seems to be beneficial, especially in alleviating troublesome pruritus [189].

### Phosphodiesterase (PDE)-4 inhibitors

PDE is a crucial regulator of cytokine production, and PDE-4 is the most abundant isoenzyme found in human leukocytes [190]. In addition, T and B cells, macrophages, monocytes, neutrophils, and eosinophils also express PDE-4 [191]. Inhibition of PDE-4 resulted in an accumulation of intracellular cyclic adenosine monophosphate (cAMP), which in turn inhibits proinflammatory cytokine transcription and production [192]. It is also known that AD mononuclear leukocytes exhibit increased cAMP-PDE activity that leads to inflammation [193].

PDE-4 inhibitors have been developed in both topical and orally administered forms. Clinical trials evaluating the efficacy of these drugs are detailed in Table 1. Topical PDE-4 inhibitors, e.g., crisaborole [194] and E6005 [195], demonstrate clinical efficacy for AD in phase II and III clinical trials, respectively. Moreover, apremilast, an oral PDE-4 inhibitor that has been used in various inflammatory diseases, including psoriasis, has shown promising results for AD in a recent phase II study [196].

### Chemoattractant receptor-homologous molecule expressed on Th2 lymphocytes (CRTh2)

CRTh2 is a prostaglandin D2 receptor that is expressed in Th2 cells, ILC2, eosinophils, and basophils [197, 198]. Activation of CRTh2 induces Th2 cell, ILC2, and eosinophil chemotaxis and promotes their cytokine production [197, 199]. CRTh2 inhibitors are currently in clinical trials for allergic diseases such as asthma and AD. For AD, the results are not yet available (NCT01785602 and NCT02002208).

### JAK inhibitor

The JAK-STAT signaling pathway involves a family of cytoplasmic protein tyrosine kinases that is essential for the induction of the cellular responses of many pivotal pathogenic cytokines in AD, namely, IL-4/IL-13 [103]. Furthermore, the activation of eosinophils, the maturation of B cells, and the suppression of regulatory T cells (Tregs) are mediated through the JAK-STAT signaling pathway [200]. Indeed, JTE052, a JAK inhibitor, has been shown to decrease STAT3 activation and leads to an improvement in the skin barrier and an upregulation of filaggrin in a murine AD model [103]. Moreover, the JAK inhibitor can inhibit IFN-γ, IL-13, and IL-17A production from antigen-specific T cells and decrease differentiation and proliferation of effector memory T cells in the draining lymph nodes in the sensitization phase of a murine contact hypersensitivity (CHS) model [201]. Therefore, it appears to be promising as a treatment of various inflammatory dermatoses. JAK inhibitors have been investigated as a treatment for psoriasis and alopecia totalis [202]. In addition, a greater percentage change from baseline (a reduction) in the EASI score was observed in a phase II clinical trial involving topical tofacitinib [203]. Additional studies are currently underway (Table 1).

### TSLP antagonist

As mentioned earlier, TSLP is crucial for Th2 skewing and is of great importance in inflammation and itching in AD. This makes TSLP as a promising target molecule to improve AD. Tezepelumab, its antagonist, is currently being studied (NCT02525094).

Other possible biologics for AD that have been studied include drugs that target IgE and B cells, as an increase in B cells and IgE is commonly observed in AD patients [204]. But the outcomes of the treatment with omalizumab, a humanized, monoclonal anti-IgE antibody, in multiple case reports and few control trials have appeared inconclusive [205–207].

In addition, rituximab, a chimeric monoclonal antibody against CD20 that depletes immature and mature B cells, also shows conflicting results [208–210]. Further investigation of omalizumab and rituximab for this indication has therefore been discontinued. Ligelizumab, a new anti-IgE antibody that exhibits a stronger IgE inhibitory effect compared to that of omalizumab [211] may, however, be beneficial for AD and is currently in a phase II trial (NCT01552629).

In summary, there are several promising therapies in the pipeline to satisfy the unmet needs of AD treatment [212]. Further insights into the disease mechanisms will tremendously improve the treatment outcomes and improve the quality of life for individuals with AD.

## Conclusions

The skin barrier, innate and adaptive immunity, and pruritus mutually orchestrate skin inflammation in AD. The pathogenesis of the disease is complex and many aspects require further clarification. Based on the detailed evidence available to date, certain disease mechanisms can be chosen as treatment targets. Many clinical trials of biological agents are currently being conducted. These new drugs in the pipeline may satisfy the unmet needs in the treatment of AD.

## Abbreviations

AD: Atopic dermatitis
CRTh2: Chemoattractant receptor-homologous molecule expressed on Th2 lymphocytes
DCs: Dendritic cells
EASI: Eczema Area and Severity Index
FLG: Filaggrin
ILCs: Innate lymphoid cells
ISGA: Investigator’s Static Global Assessment
JAK: Janus kinase
LCs: Langerhans cells
pDC: Plasmacytoid DC
PDE: Phosphodiesterase
SC: Stratum corneum
SCORAD: SCORing Atopic Dermatitis
SG: Stratum granulosum
STAT: Signal transducer and activator of transcription
TARC: Thymus- and activation-regulated chemokine (also known as CCL17)
TJs: Tight junctions
TLR: Toll-like receptor
TSLP: Thymic stromal lymphopoietin

## References

[1] Weidinger S, Novak N (2016). Atopic dermatitis. Lancet.

[2] Wallach D, Taieb A (2014). Atopic dermatitis/atopic eczema. Chem Immunol Allergy.

[3] Beattie PE, Lewis-Jones MS (2006). A comparative study of impairment of quality of life in children with skin disease and children with other chronic childhood diseases. Br J Dermatol.

[4] Holm JG, Agner T, Clausen ML, Thomsen SF (2016). Quality of life and disease severity in patients with atopic dermatitis. J Eur Acad Dermatol Venereol.

[5] Bieber T (2008). Atopic dermatitis. N Engl J Med.

[6] Alduraywish SA, Lodge CJ, Campbell B, Allen KJ, Erbas B, Lowe AJ (2016). The march from early life food sensitization to allergic disease: a systematic review and meta-analyses of birth cohort studies. Allergy.

[7] Saunders SP, Moran T, Floudas A, Wurlod F, Kaszlikowska A, Salimi M (2016). Spontaneous atopic dermatitis is mediated by innate immunity, with the secondary lung inflammation of the atopic march requiring adaptive immunity. J Allergy Clin Immunol.

[8] Lee HJ, Lee NR, Kim BK, Jung M, Kim DH, Moniaga CS (2017). Acidification of stratum corneum prevents the progression from atopic dermatitis to respiratory allergy. Exp Dermatol.

[9] Brunner PM, Silverberg JI, Guttman-Yassky E, Paller AS, Kabashima K, Amagai M (2017). Increasing comorbidities suggest that atopic dermatitis is a systemic disorder. J Invest Dermatol.

[10] Nomura T, Kayama T, Okamura E, Ogino K, Uji A, Yoshimura N (2013). Severe atopic dermatitis accompanied by autoimmune retinopathy. Eur J Dermatol.

[11] Werfel T, Allam JP, Biedermann T, Eyerich K, Gilles S, Guttman-Yassky E (2016). Cellular and molecular immunologic mechanisms in patients with atopic dermatitis. J Allergy Clin Immunol.

[12] Illi S, von Mutius E, Lau S, Nickel R, Gruber C, Niggemann B (2004). The natural course of atopic dermatitis from birth to age 7 years and the association with asthma. J Allergy Clin Immunol.

[13] Margolis JS, Abuabara K, Bilker W, Hoffstad O, Margolis DJ (2014). Persistence of mild to moderate atopic dermatitis. JAMA Dermatol.

[14] Mortz CG, Andersen KE, Dellgren C, Barington T, Bindslev-Jensen C (2015). Atopic dermatitis from adolescence to adulthood in the TOACS cohort: prevalence, persistence and comorbidities. Allergy.

[15] Kabashima K, Otsuka A, Nomura T (2016). Linking air pollution to atopic dermatitis. Nat Immunol.

[16] Dainichi T, Hanakawa S, Kabashima K (2014). Classification of inflammatory skin diseases: a proposal based on the disorders of the three-layered defense systems, barrier, innate immunity and acquired immunity. J Dermatol Sci.

[17] Kashiwakura J, Okayama Y, Furue M, Kabashima K, Shimada S, Ra C (2012). Most highly cytokinergic IgEs have polyreactivity to autoantigens. Allergy Asthma Immunol Res.

[18] Egawa G, Kabashima K (2016). Multifactorial skin barrier deficiency and atopic dermatitis: essential topics to prevent the atopic march. J Allergy Clin Immunol.

[19] Kirschner N, Houdek P, Fromm M, Moll I, Brandner JM (2010). Tight junctions form a barrier in human epidermis. Eur J Cell Biol.

[20] Steinert PM, Cantieri JS, Teller DC, Lonsdale-Eccles JD, Dale BA (1981). Characterization of a class of cationic proteins that specifically interact with intermediate filaments. Proc Natl Acad Sci U S A.

[21] Leyvraz C, Charles RP, Rubera I, Guitard M, Rotman S, Breiden B (2005). The epidermal barrier function is dependent on the serine protease CAP1/Prss8. J Cell Biol.

[22] Matsui T, Miyamoto K, Kubo A, Kawasaki H, Ebihara T, Hata K (2011). SASPase regulates stratum corneum hydration through profilaggrin-to-filaggrin processing. EMBO Mol Med.

[23] Egawa G, Doi H, Miyachi Y, Kabashima K (2013). Skin tape stripping and cheek swab method for a detection of filaggrin. J Dermatol Sci.

[24] Hoste E, Kemperman P, Devos M, Denecker G, Kezic S, Yau N (2011). Caspase-14 is required for filaggrin degradation to natural moisturizing factors in the skin. J Invest Dermatol.

[25] Kamata Y, Taniguchi A, Yamamoto M, Nomura J, Ishihara K, Takahara H (2009). Neutral cysteine protease bleomycin hydrolase is essential for the breakdown of deiminated filaggrin into amino acids. J Biol Chem.

[26] Brandner JM, Kief S, Grund C, Rendl M, Houdek P, Kuhn C (2002). Organization and formation of the tight junction system in human epidermis and cultured keratinocytes. Eur J Cell Biol.

[27] Niessen CM (2007). Tight junctions/adherens junctions: basic structure and function. J Invest Dermatol.

[28] Furuse M, Hata M, Furuse K, Yoshida Y, Haratake A, Sugitani Y (2002). Claudin-based tight junctions are crucial for the mammalian epidermal barrier: a lesson from claudin-1-deficient mice. J Cell Biol.

[29] De Benedetto A, Rafaels NM, McGirt LY, Ivanov AI, Georas SN, Cheadle C (2011). Tight junction defects in patients with atopic dermatitis. J Allergy Clin Immunol.

[30] Yuki T, Tobiishi M, Kusaka-Kikushima A, Ota Y, Tokura Y (2016). Impaired tight junctions in atopic dermatitis skin and in a skin-equivalent model treated with interleukin-17. PLoS One.

[31] Brown SJ, McLean WH (2012). One remarkable molecule: filaggrin. J Invest Dermatol.

[32] Thyssen JP, Kezic S (2014). Causes of epidermal filaggrin reduction and their role in the pathogenesis of atopic dermatitis. J Allergy Clin Immunol.

[33] Pendaries V, Malaisse J, Pellerin L, Le Lamer M, Nachat R, Kezic S (2014). Knockdown of filaggrin in a three-dimensional reconstructed human epidermis impairs keratinocyte differentiation. J Invest Dermatol.

[34] Palmer CN, Irvine AD, Terron-Kwiatkowski A, Zhao Y, Liao H, Lee SP (2006). Common loss-of-function variants of the epidermal barrier protein filaggrin are a major predisposing factor for atopic dermatitis. Nat Genet.

[35] Paternoster L, Standl M, Waage J, Baurecht H, Hotze M, Strachan DP (2015). Multi-ancestry genome-wide association study of 21,000 cases and 95,000 controls identifies new risk loci for atopic dermatitis. Nat Genet.

[36] Irvine AD, McLean WH, Leung DY (2011). Filaggrin mutations associated with skin and allergic diseases. N Engl J Med.

[37] Brattsand M, Stefansson K, Lundh C, Haasum Y, Egelrud T (2005). A proteolytic cascade of kallikreins in the stratum corneum. J Invest Dermatol.

[38] Kezic S, O’Regan GM, Lutter R, Jakasa I, Koster ES, Saunders S (2012). Filaggrin loss-of-function mutations are associated with enhanced expression of IL-1 cytokines in the stratum corneum of patients with atopic dermatitis and in a murine model of filaggrin deficiency. J Allergy Clin Immunol.

[39] Briot A, Deraison C, Lacroix M, Bonnart C, Robin A, Besson C (2009). Kallikrein 5 induces atopic dermatitis-like lesions through PAR2-mediated thymic stromal lymphopoietin expression in Netherton syndrome. J Exp Med.

[40] Moniaga CS, Jeong SK, Egawa G, Nakajima S, Hara-Chikuma M, Jeon JE (2013). Protease activity enhances production of thymic stromal lymphopoietin and basophil accumulation in flaky tail mice. Am J Pathol.

[41] Moniaga CS, Egawa G, Kawasaki H, Hara-Chikuma M, Honda T, Tanizaki H (2010). Flaky tail mouse denotes human atopic dermatitis in the steady state and by topical application with Dermatophagoides pteronyssinus extract. Am J Pathol.

[42] Moniaga CS, Kabashima K (2011). Filaggrin in atopic dermatitis: flaky tail mice as a novel model for developing drug targets in atopic dermatitis. Inflamm Allergy Drug Targets.

[43] Ewald DA, Noda S, Oliva M, Litman T, Nakajima S, Li X, et al. Major differences between human atopic dermatitis and murine models, as determined by using global transcriptomic profiling. J Allergy Clin Immunol. 2017;139(2):562–71. 10.1016/j.jaci.2016.08.029 27702671

[44] Sasaki T, Shiohama A, Kubo A, Kawasaki H, Ishida-Yamamoto A, Yamada T (2013). A homozygous nonsense mutation in the gene for Tmem79, a component for the lamellar granule secretory system, produces spontaneous eczema in an experimental model of atopic dermatitis. J Allergy Clin Immunol.

[45] Kawasaki H, Nagao K, Kubo A, Hata T, Shimizu A, Mizuno H (2012). Altered stratum corneum barrier and enhanced percutaneous immune responses in filaggrin-null mice. J Allergy Clin Immunol.

[46] Fallon PG, Sasaki T, Sandilands A, Campbell LE, Saunders SP, Mangan NE (2009). A homozygous frameshift mutation in the mouse Flg gene facilitates enhanced percutaneous allergen priming. Nat Genet.

[47] Jeong SK, Kim HJ, Youm JK, Ahn SK, Choi EH, Sohn MH (2008). Mite and cockroach allergens activate protease-activated receptor 2 and delay epidermal permeability barrier recovery. J Invest Dermatol.

[48] Miajlovic H, Fallon PG, Irvine AD, Foster TJ (2010). Effect of filaggrin breakdown products on growth of and protein expression by Staphylococcus aureus. J Allergy Clin Immunol.

[49] Brauweiler AM, Bin L, Kim BE, Oyoshi MK, Geha RS, Goleva E (2013). Filaggrin-dependent secretion of sphingomyelinase protects against staphylococcal alpha-toxin-induced keratinocyte death. J Allergy Clin Immunol.

[50] Mildner M, Jin J, Eckhart L, Kezic S, Gruber F, Barresi C (2010). Knockdown of filaggrin impairs diffusion barrier function and increases UV sensitivity in a human skin model. J Invest Dermatol.

[51] Scharschmidt TC, Man MQ, Hatano Y, Crumrine D, Gunathilake R, Sundberg JP (2009). Filaggrin deficiency confers a paracellular barrier abnormality that reduces inflammatory thresholds to irritants and hapten. J Allergy Clin Immunol.

[52] Otsuka A, Doi H, Egawa G, Maekawa A, Fujita T, Nakamizo S (2014). Possible new therapeutic strategy to regulate atopic dermatitis through upregulating filaggrin expression. J Allergy Clin Immunol.

[53] Matsui S, Murota H, Takahashi A, Yang L, Lee JB, Omiya K (2014). Dynamic analysis of histamine-mediated attenuation of acetylcholine-induced sweating via GSK3beta activation. J Invest Dermatol.

[54] Murota H, Matsui S, Ono E, Kijima A, Kikuta J, Ishii M (2015). Sweat, the driving force behind normal skin: an emerging perspective on functional biology and regulatory mechanisms. J Dermatol Sci.

[55] Rerknimitr P, Tanizaki H, Yamamoto Y, Amano W, Nakajima S, Nakashima C, et al. Decreased Filaggrin Level May Lead to Sweat Duct Obstruction in Filaggrin Mutant Mice. J Invest Dermatol. 2017;137(1):248–51. 10.1016/j.jid.2016.07.036 27591778

[56] Eckl KM, de Juanes S, Kurtenbach J, Natebus M, Lugassy J, Oji V (2009). Molecular analysis of 250 patients with autosomal recessive congenital ichthyosis: evidence for mutation hotspots in ALOXE3 and allelic heterogeneity in ALOX12B. J Invest Dermatol.

[57] Saunders SP, Goh CS, Brown SJ, Palmer CN, Porter RM, Cole C (2013). Tmem79/Matt is the matted mouse gene and is a predisposing gene for atopic dermatitis in human subjects. J Allergy Clin Immunol.

[58] Deraison C, Bonnart C, Lopez F, Besson C, Robinson R, Jayakumar A (2007). LEKTI fragments specifically inhibit KLK5, KLK7, and KLK14 and control desquamation through a pH-dependent interaction. Mol Biol Cell.

[59] Vasilopoulos Y, Sharaf N, di Giovine F, Simon M, Cork MJ, Duff GW (2011). The 3’-UTR AACCins5874 in the stratum corneum chymotryptic enzyme gene (SCCE/KLK7), associated with atopic dermatitis; causes an increased mRNA expression without altering its stability. J Dermatol Sci.

[60] Chavanas S, Bodemer C, Rochat A, Hamel-Teillac D, Ali M, Irvine AD (2000). Mutations in SPINK5, encoding a serine protease inhibitor, cause Netherton syndrome. Nat Genet.

[61] Walley AJ, Chavanas S, Moffatt MF, Esnouf RM, Ubhi B, Lawrence R (2001). Gene polymorphism in Netherton and common atopic disease. Nat Genet.

[62] Zhao LP, Di Z, Zhang L, Wang L, Ma L, Lv Y (2012). Association of SPINK5 gene polymorphisms with atopic dermatitis in Northeast China. J Eur Acad Dermatol Venereol.

[63] Kabashima K (2013). New concept of the pathogenesis of atopic dermatitis: interplay among the barrier, allergy, and pruritus as a trinity. J Dermatol Sci.

[64] Hirota T, Takahashi A, Kubo M, Tsunoda T, Tomita K, Sakashita M (2012). Genome-wide association study identifies eight new susceptibility loci for atopic dermatitis in the Japanese population. Nat Genet.

[65] Oyoshi MK, Larson RP, Ziegler SF, Geha RS (2010). Mechanical injury polarizes skin dendritic cells to elicit a T(H)2 response by inducing cutaneous thymic stromal lymphopoietin expression. J Allergy Clin Immunol.

[66] Yoo J, Omori M, Gyarmati D, Zhou B, Aye T, Brewer A (2005). Spontaneous atopic dermatitis in mice expressing an inducible thymic stromal lymphopoietin transgene specifically in the skin. J Exp Med.

[67] Onoue A, Kabashima K, Kobayashi M, Mori T, Tokura Y (2009). Induction of eosinophil- and Th2-attracting epidermal chemokines and cutaneous late-phase reaction in tape-stripped skin. Exp Dermatol.

[68] Nakahigashi K, Kabashima K, Ikoma A, Verkman AS, Miyachi Y, Hara-Chikuma M (2011). Upregulation of aquaporin-3 is involved in keratinocyte proliferation and epidermal hyperplasia. J Invest Dermatol.

[69] Kim BS (2015). Innate lymphoid cells in the skin. J Invest Dermatol.

[70] Bruggen MC, Bauer WM, Reininger B, Clim E, Captarencu C, Steiner GE, et al. In Situ Mapping of Innate Lymphoid Cells in Human Skin: Evidence for Remarkable Differences between Normal and Inflamed Skin. J Invest Dermatol. 2016;136(12):2396–405. 10.1016/j.jid.2016.07.017 27456756

[71] Kim BS, Siracusa MC, Saenz SA, Noti M, Monticelli LA, Sonnenberg GF (2013). TSLP elicits IL-33-independent innate lymphoid cell responses to promote skin inflammation. Sci Transl Med.

[72] Roediger B, Kyle R, Yip KH, Sumaria N, Guy TV, Kim BS (2013). Cutaneous immunosurveillance and regulation of inflammation by group 2 innate lymphoid cells. Nat Immunol.

[73] Salimi M, Barlow JL, Saunders SP, Xue L, Gutowska-Owsiak D, Wang X (2013). A role for IL-25 and IL-33-driven type-2 innate lymphoid cells in atopic dermatitis. J Exp Med.

[74] Imai Y, Yasuda K, Sakaguchi Y, Haneda T, Mizutani H, Yoshimoto T (2013). Skin-specific expression of IL-33 activates group 2 innate lymphoid cells and elicits atopic dermatitis-like inflammation in mice. Proc Natl Acad Sci U S A.

[75] Xue L, Salimi M, Panse I, Mjosberg JM, McKenzie AN, Spits H (2014). Prostaglandin D2 activates group 2 innate lymphoid cells through chemoattractant receptor-homologous molecule expressed on TH2 cells. J Allergy Clin Immunol.

[76] Honda T, Kabashima K (2015). Prostanoids in allergy. Allergol Int.

[77] Kim BS, Wang K, Siracusa MC, Saenz SA, Brestoff JR, Monticelli LA (2014). Basophils promote innate lymphoid cell responses in inflamed skin. J Immunol.

[78] Nakai K, Yoneda K, Hosokawa Y, Moriue T, Presland RB, Fallon PG (2012). Reduced expression of epidermal growth factor receptor, E-cadherin, and occludin in the skin of flaky tail mice is due to filaggrin and loricrin deficiencies. Am J Pathol.

[79] Morita H, Moro K, Koyasu S (2016). Innate lymphoid cells in allergic and nonallergic inflammation. J Allergy Clin Immunol.

[80] Otsuka A, Nonomura Y, Kabashima K (2016). Roles of basophils and mast cells in cutaneous inflammation. Semin Immunopathol.

[81] Otsuka A, Kabashima K (2015). Mast cells and basophils in cutaneous immune responses. Allergy.

[82] Nakashima C, Otsuka A, Kitoh A, Honda T, Egawa G, Nakajima S (2014). Basophils regulate the recruitment of eosinophils in a murine model of irritant contact dermatitis. J Allergy Clin Immunol.

[83] Mori T, Kabashima K, Fukamachi S, Kuroda E, Sakabe J, Kobayashi M (2013). D1-like dopamine receptors antagonist inhibits cutaneous immune reactions mediated by Th2 and mast cells. J Dermatol Sci.

[84] Gros E, Novak N (2012). Cutaneous dendritic cells in allergic inflammation. Clin Exp Allergy.

[85] Kubo A, Nagao K, Yokouchi M, Sasaki H, Amagai M (2009). External antigen uptake by Langerhans cells with reorganization of epidermal tight junction barriers. J Exp Med.

[86] Klechevsky E, Morita R, Liu M, Cao Y, Coquery S, Thompson-Snipes L (2008). Functional specializations of human epidermal Langerhans cells and CD14+ dermal dendritic cells. Immunity.

[87] Nakajima S, Igyarto BZ, Honda T, Egawa G, Otsuka A, Hara-Chikuma M (2012). Langerhans cells are critical in epicutaneous sensitization with protein antigen via thymic stromal lymphopoietin receptor signaling. J Allergy Clin Immunol.

[88] Elentner A, Finke D, Schmuth M, Chappaz S, Ebner S, Malissen B (2009). Langerhans cells are critical in the development of atopic dermatitis-like inflammation and symptoms in mice. J Cell Mol Med.

[89] Ziegler SF (2010). The role of thymic stromal lymphopoietin (TSLP) in allergic disorders. Curr Opin Immunol.

[90] Fernandez MI, Heuze ML, Martinez-Cingolani C, Volpe E, Donnadieu MH, Piel M (2011). The human cytokine TSLP triggers a cell-autonomous dendritic cell migration in confined environments. Blood.

[91] Ebner S, Nguyen VA, Forstner M, Wang YH, Wolfram D, Liu YJ (2007). Thymic stromal lymphopoietin converts human epidermal Langerhans cells into antigen-presenting cells that induce proallergic T cells. J Allergy Clin Immunol.

[92] Novak N (2012). An update on the role of human dendritic cells in patients with atopic dermatitis. J Allergy Clin Immunol.

[93] Novak N, Valenta R, Bohle B, Laffer S, Haberstok J, Kraft S (2004). FcepsilonRI engagement of Langerhans cell-like dendritic cells and inflammatory dendritic epidermal cell-like dendritic cells induces chemotactic signals and different T-cell phenotypes in vitro. J Allergy Clin Immunol.

[94] Kerschenlohr K, Decard S, Przybilla B, Wollenberg A (2003). Atopy patch test reactions show a rapid influx of inflammatory dendritic epidermal cells in patients with extrinsic atopic dermatitis and patients with intrinsic atopic dermatitis. J Allergy Clin Immunol.

[95] Wollenberg A, Wagner M, Gunther S, Towarowski A, Tuma E, Moderer M (2002). Plasmacytoid dendritic cells: a new cutaneous dendritic cell subset with distinct role in inflammatory skin diseases. J Invest Dermatol.

[96] Albanesi C, Scarponi C, Pallotta S, Daniele R, Bosisio D, Madonna S (2009). Chemerin expression marks early psoriatic skin lesions and correlates with plasmacytoid dendritic cell recruitment. J Exp Med.

[97] Novak N, Allam JP, Hagemann T, Jenneck C, Laffer S, Valenta R (2004). Characterization of FcepsilonRI-bearing CD123 blood dendritic cell antigen-2 plasmacytoid dendritic cells in atopic dermatitis. J Allergy Clin Immunol.

[98] Moy AP, Murali M, Kroshinsky D, Duncan LM, Nazarian RM (2015). Immunologic overlap of helper T-cell subtypes 17 and 22 in erythrodermic psoriasis and atopic dermatitis. JAMA Dermatol.

[99] Nygaard U, Hvid M, Johansen C, Buchner M, Folster-Holst R, Deleuran M (2016). TSLP, IL-31, IL-33 and sST2 are new biomarkers in endophenotypic profiling of adult and childhood atopic dermatitis. J Eur Acad Dermatol Venereol.

[100] Kim JE, Kim JS, Cho DH, Park HJ. Molecular mechanisms of cutaneous inflammatory disorder: atopic dermatitis. Int J Mol Sci. 2016;17. 10.3390/ijms17081234 PMC5000632 27483258

[101] Howell MD, Kim BE, Gao P, Grant AV, Boguniewicz M, Debenedetto A (2007). Cytokine modulation of atopic dermatitis filaggrin skin expression. J Allergy Clin Immunol.

[102] Kim BE, Leung DY, Boguniewicz M, Howell MD (2008). Loricrin and involucrin expression is down-regulated by Th2 cytokines through STAT-6. Clin Immunol.

[103] Amano W, Nakajima S, Kunugi H, Numata Y, Kitoh A, Egawa G (2015). The Janus kinase inhibitor JTE-052 improves skin barrier function through suppressing signal transducer and activator of transcription 3 signaling. J Allergy Clin Immunol.

[104] Mitsuishi T, Kabashima K, Tanizaki H, Ohsawa I, Oda F, Yamada Y (2011). Specific substance of Maruyama (SSM) suppresses immune responses in atopic dermatitis-like skin lesions in DS-Nh mice by modulating dendritic cell functions. J Dermatol Sci.

[105] Watcharanurak K, Nishikawa M, Takahashi Y, Kabashima K, Takahashi R, Takakura Y (2013). Regulation of immunological balance by sustained interferon-gamma gene transfer for acute phase of atopic dermatitis in mice. Gene Ther.

[106] Hattori K, Nishikawa M, Watcharanurak K, Ikoma A, Kabashima K, Toyota H (2010). Sustained exogenous expression of therapeutic levels of IFN-gamma ameliorates atopic dermatitis in NC/Nga mice via Th1 polarization. J Immunol.

[107] Thaçi D, Simpson EL, Beck LA, Bieber T, Blauvelt A, Papp K, et al. Efficacy and safety of dupilumab in adults with moderate-to-severe atopic dermatitis inadequately controlled by topical treatments: a randomised, placebo-controlled, dose-ranging phase 2b trial. Lancet. 2016;387(10013):40–52. 10.1016/S0140-6736(15)00388-8 26454361

[108] Nomura T, Kabashima K, Miyachi Y (2014). The panoply of alphabetaT cells in the skin. J Dermatol Sci.

[109] Jin S, Park CO, Shin JU, Noh JY, Lee YS, Lee NR (2014). DAMP molecules S100A9 and S100A8 activated by IL-17A and house-dust mites are increased in atopic dermatitis. Exp Dermatol.

[110] Park H, Li Z, Yang XO, Chang SH, Nurieva R, Wang YH (2005). A distinct lineage of CD4 T cells regulates tissue inflammation by producing interleukin 17. Nat Immunol.

[111] He R, Oyoshi MK, Jin H, Geha RS (2007). Epicutaneous antigen exposure induces a Th17 response that drives airway inflammation after inhalation challenge. Proc Natl Acad Sci U S A.

[112] Nakajima S, Kitoh A, Egawa G, Natsuaki Y, Nakamizo S, Moniaga CS (2014). IL-17A as an inducer for Th2 immune responses in murine atopic dermatitis models. J Invest Dermatol.

[113] Oyoshi MK, Murphy GF, Geha RS (2009). Filaggrin-deficient mice exhibit TH17-dominated skin inflammation and permissiveness to epicutaneous sensitization with protein antigen. J Allergy Clin Immunol.

[114] Toda M, Leung DY, Molet S, Boguniewicz M, Taha R, Christodoulopoulos P (2003). Polarized in vivo expression of IL-11 and IL-17 between acute and chronic skin lesions. J Allergy Clin Immunol.

[115] Koga C, Kabashima K, Shiraishi N, Kobayashi M, Tokura Y (2008). Possible pathogenic role of Th17 cells for atopic dermatitis. J Invest Dermatol.

[116] Tokura Y (2010). Extrinsic and intrinsic types of atopic dermatitis. J Dermatol Sci.

[117] Kabashima-Kubo R, Nakamura M, Sakabe J, Sugita K, Hino R, Mori T (2012). A group of atopic dermatitis without IgE elevation or barrier impairment shows a high Th1 frequency: possible immunological state of the intrinsic type. J Dermatol Sci.

[118] Mori T, Ishida K, Mukumoto S, Yamada Y, Imokawa G, Kabashima K (2010). Comparison of skin barrier function and sensory nerve electric current perception threshold between IgE-high extrinsic and IgE-normal intrinsic types of atopic dermatitis. Br J Dermatol.

[119] Suarez-Farinas M, Dhingra N, Gittler J, Shemer A, Cardinale I, de Guzman SC (2013). Intrinsic atopic dermatitis shows similar TH2 and higher TH17 immune activation compared with extrinsic atopic dermatitis. J Allergy Clin Immunol.

[120] Noda S, Suarez-Farinas M, Ungar B, Kim SJ, de Guzman SC, Xu H (2015). The Asian atopic dermatitis phenotype combines features of atopic dermatitis and psoriasis with increased TH17 polarization. J Allergy Clin Immunol.

[121] Kong HH, Oh J, Deming C, Conlan S, Grice EA, Beatson MA (2012). Temporal shifts in the skin microbiome associated with disease flares and treatment in children with atopic dermatitis. Genome Res.

[122] Harder J, Schroder JM, Glaser R (2013). The skin surface as antimicrobial barrier: present concepts and future outlooks. Exp Dermatol.

[123] Ong PY, Ohtake T, Brandt C, Strickland I, Boguniewicz M, Ganz T (2002). Endogenous antimicrobial peptides and skin infections in atopic dermatitis. N Engl J Med.

[124] Glaser R, Meyer-Hoffert U, Harder J, Cordes J, Wittersheim M, Kobliakova J (2009). The antimicrobial protein psoriasin (S100A7) is upregulated in atopic dermatitis and after experimental skin barrier disruption. J Invest Dermatol.

[125] Harder J, Dressel S, Wittersheim M, Cordes J, Meyer-Hoffert U, Mrowietz U (2010). Enhanced expression and secretion of antimicrobial peptides in atopic dermatitis and after superficial skin injury. J Invest Dermatol.

[126] Schittek B (2011). The antimicrobial skin barrier in patients with atopic dermatitis. Curr Probl Dermatol.

[127] Son ED, Kim HJ, Kim KH, Bin BH, Bae IH, Lim KM (2016). S100A7 (psoriasin) inhibits human epidermal differentiation by enhanced IL-6 secretion through IkappaB/NF-kappaB signalling. Exp Dermatol.

[128] Fornasa G, Tsilingiri K, Caprioli F, Botti F, Mapelli M, Meller S (2015). Dichotomy of short and long thymic stromal lymphopoietin isoforms in inflammatory disorders of the bowel and skin. J Allergy Clin Immunol.

[129] Bjerkan L, Schreurs O, Engen SA, Jahnsen FL, Baekkevold ES, Blix IJ (2015). The short form of TSLP is constitutively translated in human keratinocytes and has characteristics of an antimicrobial peptide. Mucosal Immunol.

[130] Bjerkan L, Sonesson A, Schenck K. Multiple functions of the new cytokine-based antimicrobial peptide thymic stromal lymphopoietin (TSLP). Pharmaceuticals (Basel). 2016;9. 10.3390/ph9030041 PMC5039494 27399723

[131] Kupper TS, Fuhlbrigge RC (2004). Immune surveillance in the skin: mechanisms and clinical consequences. Nat Rev Immunol.

[132] Stoll H, Dengjel J, Nerz C, Gotz F (2005). Staphylococcus aureus deficient in lipidation of prelipoproteins is attenuated in growth and immune activation. Infect Immun.

[133] Skabytska Y, Kaesler S, Volz T, Biedermann T (2016). The role of innate immune signaling in the pathogenesis of atopic dermatitis and consequences for treatments. Semin Immunopathol.

[134] Ahmad-Nejad P, Mrabet-Dahbi S, Breuer K, Klotz M, Werfel T, Herz U (2004). The toll-like receptor 2 R753Q polymorphism defines a subgroup of patients with atopic dermatitis having severe phenotype. J Allergy Clin Immunol.

[135] Mrabet-Dahbi S, Dalpke AH, Niebuhr M, Frey M, Draing C, Brand S (2008). The Toll-like receptor 2 R753Q mutation modifies cytokine production and Toll-like receptor expression in atopic dermatitis. J Allergy Clin Immunol.

[136] Voss E, Wehkamp J, Wehkamp K, Stange EF, Schroder JM, Harder J (2006). NOD2/CARD15 mediates induction of the antimicrobial peptide human beta-defensin-2. J Biol Chem.

[137] Hruz P, Zinkernagel AS, Jenikova G, Botwin GJ, Hugot JP, Karin M (2009). NOD2 contributes to cutaneous defense against Staphylococcus aureus through alpha-toxin-dependent innate immune activation. Proc Natl Acad Sci U S A.

[138] Macaluso F, Nothnagel M, Parwez Q, Petrasch-Parwez E, Bechara FG, Epplen JT (2007). Polymorphisms in NACHT-LRR (NLR) genes in atopic dermatitis. Exp Dermatol.

[139] Volz T, Nega M, Buschmann J, Kaesler S, Guenova E, Peschel A (2010). Natural Staphylococcus aureus-derived peptidoglycan fragments activate NOD2 and act as potent costimulators of the innate immune system exclusively in the presence of TLR signals. Faseb J.

[140] Miller LS, Cho JS (2011). Immunity against Staphylococcus aureus cutaneous infections. Nat Rev Immunol.

[141] Islander U, Andersson A, Lindberg E, Adlerberth I, Wold AE, Rudin A (2010). Superantigenic Staphylococcus aureus stimulates production of interleukin-17 from memory but not naive T cells. Infect Immun.

[142] Czarnowicki T, Gonzalez J, Shemer A, Malajian D, Xu H, Zheng X (2015). Severe atopic dermatitis is characterized by selective expansion of circulating TH2/TC2 and TH22/TC22, but not TH17/TC17, cells within the skin-homing T-cell population. J Allergy Clin Immunol.

[143] Hepburn L, Hijnen DJ, Sellman BR, Mustelin T, Sleeman MA, May RD, et al. The complex biology and contribution of Staphylococcus aureus in atopic dermatitis, current and future therapies. Br J Dermatol. 2016. doi:10.1111/bjd.15139. [Epub ahead of print]. 10.1111/bjd.15139 27779765

[144] Hirasawa Y, Takai T, Nakamura T, Mitsuishi K, Gunawan H, Suto H (2010). Staphylococcus aureus extracellular protease causes epidermal barrier dysfunction. J Invest Dermatol.

[145] Takeuchi O, Akira S (2010). Pattern recognition receptors and inflammation. Cell.

[146] Spaulding AR, Satterwhite EA, Lin YC, Chuang-Smith ON, Frank KL, Merriman JA (2012). Comparison of Staphylococcus aureus strains for ability to cause infective endocarditis and lethal sepsis in rabbits. Front Cell Infect Microbiol.

[147] Leung DY, Travers JB, Giorno R, Norris DA, Skinner R, Aelion J (1995). Evidence for a streptococcal superantigen-driven process in acute guttate psoriasis. J Clin Invest.

[148] Leung DY, Harbeck R, Bina P, Reiser RF, Yang E, Norris DA (1993). Presence of IgE antibodies to staphylococcal exotoxins on the skin of patients with atopic dermatitis. Evidence for a new group of allergens. J Clin Invest.

[149] Nakamura Y, Oscherwitz J, Cease KB, Chan SM, Munoz-Planillo R, Hasegawa M (2013). Staphylococcus delta-toxin induces allergic skin disease by activating mast cells. Nature.

[150] Nakamizo S, Egawa G, Honda T, Nakajima S, Belkaid Y, Kabashima K (2015). Commensal bacteria and cutaneous immunity. Semin Immunopathol.

[151] Kido-Nakahara M, Furue M, Ulzii D, Nakahara T (2017). Itch in atopic dermatitis. Immunol Allergy Clin North Am.

[152] Kamo A, Tominaga M, Tengara S, Ogawa H, Takamori K (2011). Inhibitory effects of UV-based therapy on dry skin-inducible nerve growth in acetone-treated mice. J Dermatol Sci.

[153] Urashima R, Mihara M (1998). Cutaneous nerves in atopic dermatitis. A histological, immunohistochemical and electron microscopic study. Virchows Arch.

[154] Tominaga M, Tengara S, Kamo A, Ogawa H, Takamori K (2009). Psoralen-ultraviolet A therapy alters epidermal Sema3A and NGF levels and modulates epidermal innervation in atopic dermatitis. J Dermatol Sci.

[155] Toyoda M, Nakamura M, Makino T, Hino T, Kagoura M, Morohashi M (2002). Nerve growth factor and substance P are useful plasma markers of disease activity in atopic dermatitis. Br J Dermatol.

[156] Tominaga M, Ogawa H, Takamori K (2008). Decreased production of semaphorin 3A in the lesional skin of atopic dermatitis. Br J Dermatol.

[157] Mollanazar NK, Smith PK, Yosipovitch G (2016). Mediators of chronic pruritus in atopic dermatitis: getting the itch out?. Clin Rev Allergy Immunol.

[158] Han L, Dong X (2014). Itch mechanisms and circuits. Annu Rev Biophys.

[159] Ikoma A, Steinhoff M, Stander S, Yosipovitch G, Schmelz M (2006). The neurobiology of itch. Nat Rev Neurosci.

[160] Murota H, Katayama I (2016). Evolving understanding on the aetiology of thermally provoked itch. Eur J Pain.

[161] Murota H, Izumi M, Abd El-Latif MIA, Nishioka M, Terao M, Tani M (2012). Artemin causes hypersensitivity to warm sensation, mimicking warmth-provoked pruritus in atopic dermatitis. J Allergy Clin Immunol.

[162] Cowden JM, Zhang M, Dunford PJ, Thurmond RL (2010). The histamine H4 receptor mediates inflammation and pruritus in Th2-dependent dermal inflammation. J Invest Dermatol.

[163] Sonkoly E, Muller A, Lauerma AI, Pivarcsi A, Soto H, Kemeny L (2006). IL-31: a new link between T cells and pruritus in atopic skin inflammation. J Allergy Clin Immunol.

[164] Dillon SR, Sprecher C, Hammond A, Bilsborough J, Rosenfeld-Franklin M, Presnell SR (2004). Interleukin 31, a cytokine produced by activated T cells, induces dermatitis in mice. Nat Immunol.

[165] Wilson SR, Thé L, Batia LM, Beattie K, Katibah GE, McClain SP (2013). The epithelial cell-derived atopic dermatitis cytokine TSLP activates neurons to induce itch. Cell.

[166] Kido-Nakahara M, Buddenkotte J, Kempkes C, Ikoma A, Cevikbas F, Akiyama T (2014). Neural peptidase endothelin-converting enzyme 1 regulates endothelin 1-induced pruritus. J Clin Invest.

[167] Heise R, Neis MM, Marquardt Y, Joussen S, Heinrich PC, Merk HF (2009). IL-31 receptor alpha expression in epidermal keratinocytes is modulated by cell differentiation and interferon gamma. J Invest Dermatol.

[168] Kato A, Fujii E, Watanabe T, Takashima Y, Matsushita H, Furuhashi T, et al. Distribution of IL-31 and its receptor expressing cells in skin of atopic dermatitis. J Dermatol Sci. 2014;74(3):229–35. 10.1016/j.jdermsci.2014.02.009 24667097

[169] Cornelissen C, Marquardt Y, Czaja K, Wenzel J, Frank J, Lüscher-Firzlaff J (2012). IL-31 regulates differentiation and filaggrin expression in human organotypic skin models. J Allergy Clin Immunol.

[170] Kasraie S, Niebuhr M, Werfel T (2013). Interleukin (IL)-31 activates signal transducer and activator of transcription (STAT)-1, STAT-5 and extracellular signal-regulated kinase 1/2 and down-regulates IL-12p40 production in activated human macrophages. Allergy.

[171] Raap U, Wieczorek D, Gehring M, Pauls I, Ständer S, Kapp A (2010). Increased levels of serum IL-31 in chronic spontaneous urticaria. Exp Dermatol.

[172] Raap U, Wichmann K, Bruder M, Ständer S, Wedi B, Kapp A (2008). Correlation of IL-31 serum levels with severity of atopic dermatitis. J Allergy Clin Immunol.

[173] Nomura T, Kabashima K (2016). Advances in atopic dermatitis in 2015. J Allergy Clin Immunol.

[174] Ishiuji Y, Coghill RC, Patel TS, Oshiro Y, Kraft RA, Yosipovitch G (2009). Distinct patterns of brain activity evoked by histamine-induced itch reveal an association with itch intensity and disease severity in atopic dermatitis. Brit J Dermatol.

[175] Leslie TA, Greaves MW, Yosipovitch G (2015). Current topical and systemic therapies for itch. Handb Exp Pharmacol.

[176] Shiratori-Hayashi M, Koga K, Tozaki-Saitoh H, Kohro Y, Toyonaga H, Yamaguchi C (2015). STAT3-dependent reactive astrogliosis in the spinal dorsal horn underlies chronic itch. Nat Med.

[177] Tominaga M, Ozawa S, Tengara S, Ogawa H, Takamori K (2007). Intraepidermal nerve fibers increase in dry skin of acetone-treated mice. J Dermatol Sci.

[178] Heratizadeh A, Werfel T (2016). Anti-inflammatory therapies in atopic dermatitis. Allergy.

[179] Lauffer F, Ring J (2016). Target-oriented therapy: emerging drugs for atopic dermatitis. Expert Opin Emerg Drugs.

[180] Noda S, Krueger JG, Guttman-Yassky E (2015). The translational revolution and use of biologics in patients with inflammatory skin diseases. J Allergy Clin Immunol.

[181] Beck LA, Thaci D, Hamilton JD, Graham NM, Bieber T, Rocklin R (2014). Dupilumab treatment in adults with moderate-to-severe atopic dermatitis. N Engl J Med.

[182] Simpson EL, Gadkari A, Worm M, Soong W, Blauvelt A, Eckert L (2016). Dupilumab therapy provides clinically meaningful improvement in patient-reported outcomes (PROs): a phase IIb, randomized, placebo-controlled, clinical trial in adult patients with moderate to severe atopic dermatitis (AD). J Am Acad Dermatol.

[183] Hamilton JD, Suarez-Farinas M, Dhingra N, Cardinale I, Li X, Kostic A (2014). Dupilumab improves the molecular signature in skin of patients with moderate-to-severe atopic dermatitis. J Allergy Clin Immunol.

[184] Simpson EL, Bieber T, Guttman-Yassky E, Beck LA, Blauvelt A, Cork MJ (2016). Two phase 3 trials of dupilumab versus placebo in atopic dermatitis. N Engl J Med.

[185] Khattri S, Brunner PM, Garcet S, Finney R, Cohen SR, Oliva M, et al. Efficacy and safety of ustekinumab treatment in adults with moderate-to-severe atopic dermatitis. Exp Dermatol. 2017;26(1):28–35. 10.1111/exd.13112 PMC5502835 27304428

[186] Otsuka A, Tanioka M, Nakagawa Y, Honda T, Ikoma A, Miyachi Y (2011). Effects of cyclosporine on pruritus and serum IL-31 levels in patients with atopic dermatitis. Eur J Dermatol.

[187] Otsuka A, Honda T, Doi H, Miyachi Y, Kabashima K (2011). An H1-histamine receptor antagonist decreases serum interleukin-31 levels in patients with atopic dermatitis. Br J Dermatol.

[188] Kabashima K, Furue M, Hanifin J, Pulka G, Mlynarczyk I, Wollenberg A (2016). Humanized anti-interleukin-31 receptor A antibody nemolizumab (CIM331) suppresses pruritus and improves eczema in patients with moderate-to-severe atopic dermatitis. J Invest Dermatol.

[189] Nemoto O, Furue M, Nakagawa H, Shiramoto M, Hanada R, Matsuki S (2016). The first trial of CIM331, a humanized antihuman interleukin-31 receptor A antibody, in healthy volunteers and patients with atopic dermatitis to evaluate safety, tolerability and pharmacokinetics of a single dose in a randomized, double-blind, placebo-controlled study. Br J Dermatol.

[190] Chan SC, Reifsnyder D, Beavo JA, Hanifin JM (1993). Immunochemical characterization of the distinct monocyte cyclic AMP-phosphodiesterase from patients with atopic dermatitis. J Allergy Clin Immunol.

[191] Felding J, Sorensen MD, Poulsen TD, Larsen J, Andersson C, Refer P (2014). Discovery and early clinical development of 2-{6-[2-(3,5-dichloro-4-pyridyl)acetyl]-2,3-dimethoxyphenoxy}-N-propylacetamide (LEO 29102), a soft-drug inhibitor of phosphodiesterase 4 for topical treatment of atopic dermatitis. J Med Chem.

[192] Souness JE, Aldous D, Sargent C (2000). Immunosuppressive and anti-inflammatory effects of cyclic AMP phosphodiesterase (PDE) type 4 inhibitors. Immunopharmacology.

[193] Grewe SR, Chan SC, Hanifin JM (1982). Elevated leukocyte cyclic AMP-phosphodiesterase in atopic disease: a possible mechanism for cyclic AMP-agonist hyporesponsiveness. J Allergy Clin Immunol.

[194] Paller AS, Tom WL, Lebwohl MG, Blumenthal RL, Boguniewicz M, Call RS (2016). Efficacy and safety of crisaborole ointment, a novel, nonsteroidal phosphodiesterase 4 (PDE4) inhibitor for the topical treatment of atopic dermatitis (AD) in children and adults. J Am Acad Dermatol.

[195] Ohba F, Matsuki S, Imayama S, Matsuguma K, Hojo S, Nomoto M (2016). Efficacy of a novel phosphodiesterase inhibitor, E6005, in patients with atopic dermatitis: an investigator-blinded, vehicle-controlled study. J Dermatolog Treat.

[196] Volf EM, Au SC, Dumont N, Scheinman P, Gottlieb AB (2012). A phase 2, open-label, investigator-initiated study to evaluate the safety and efficacy of apremilast in subjects with recalcitrant allergic contact or atopic dermatitis. J Drugs Dermatol.

[197] Wojno ED, Monticelli LA, Tran SV, Alenghat T, Osborne LC, Thome JJ (2015). The prostaglandin D(2) receptor CRTH2 regulates accumulation of group 2 innate lymphoid cells in the inflamed lung. Mucosal Immunol.

[198] Iwasaki M, Nagata K, Takano S, Takahashi K, Ishii N, Ikezawa Z (2002). Association of a new-type prostaglandin D2 receptor CRTH2 with circulating T helper 2 cells in patients with atopic dermatitis. J Invest Dermatol.

[199] Pettipher R, Hansel TT (2008). Antagonists of the prostaglandin D2 receptor CRTH2. Drug News Perspect.

[200] Bao L, Zhang H, Chan LS (2013). The involvement of the JAK-STAT signaling pathway in chronic inflammatory skin disease atopic dermatitis. Jakstat.

[201] Amano W, Nakajima S, Yamamoto Y, Tanimoto A, Matsushita M, Miyachi Y (2016). JAK inhibitor JTE-052 regulates contact hypersensitivity by downmodulating T cell activation and differentiation. J Dermatol Sci.

[202] Samadi A, Ahmad Nasrollahi S, Hashemi A, Nassiri-Kashani M, Firooz A. Janus kinase (JAK) inhibitors for the treatment of skin and hair disorders: a review of literature. J Dermatol Treat. 2017;22:1–11. 10.1080/09546634.2016.1277179 28024126

[203] Bissonnette R, Papp KA, Poulin Y, Gooderham M, Raman M, Mallbris L (2016). Topical tofacitinib for atopic dermatitis: a phase IIa randomized trial. Brit J Dermatol.

[204] Czarnowicki T, Gonzalez J, Bonifacio KM, Shemer A, Xiangyu P, Kunjravia N (2016). Diverse activation and differentiation of multiple B-cell subsets in patients with atopic dermatitis but not in patients with psoriasis. J Allergy Clin Immunol.

[205] Krathen RA, Hsu S (2005). Failure of omalizumab for treatment of severe adult atopic dermatitis. J Am Acad Dermatol.

[206] Vigo PG, Girgis KR, Pfuetze BL, Critchlow ME, Fisher J, Hussain I (2006). Efficacy of anti-IgE therapy in patients with atopic dermatitis. J Am Acad Dermatol.

[207] Belloni B, Ziai M, Lim A, Lemercier B, Sbornik M, Weidinger S (2007). Low-dose anti-IgE therapy in patients with atopic eczema with high serum IgE levels. J Allergy Clin Immunol.

[208] Simon D, Hosli S, Kostylina G, Yawalkar N, Simon HU (2008). Anti-CD20 (rituximab) treatment improves atopic eczema. J Allergy Clin Immunol.

[209] Ponte P, Lopes MJ (2010). Apparent safe use of single dose rituximab for recalcitrant atopic dermatitis in the first trimester of a twin pregnancy. J Am Acad Dermatol.

[210] Sediva A, Kayserova J, Vernerova E, Polouckova A, Capkova S, Spisek R (2008). Anti-CD20 (rituximab) treatment for atopic eczema. J Allergy Clin Immunol.

[211] Arm JP, Bottoli I, Skerjanec A, Floch D, Groenewegen A, Maahs S (2014). Pharmacokinetics, pharmacodynamics and safety of QGE031 (ligelizumab), a novel high-affinity anti-IgE antibody, in atopic subjects. Clin Exp Allergy.

[212] Saeki H, Nakahara T, Tanaka A, Kabashima K, Sugaya M, Murota H (2016). Clinical practice guidelines for the management of atopic dermatitis 2016. J Dermatol.

